# Quantitative reflection phase mesoscopy by remote coherence tuning of phase-shift interference patterns

**DOI:** 10.1038/srep12560

**Published:** 2015-07-28

**Authors:** Elad Arbel, Alberto Bilenca

**Affiliations:** 1 Biomedical Engineering Department, Ben-Gurion University of the Negev, 1 Ben Gurion Blvd, Be’er-Sheva 8410501, Israel; 2 Ilse Katz Institute for Nanoscale Science and Technology, Ben-Gurion University of the Negev, 1 Ben Gurion Blvd, Be’er-Sheva 8410501, Israel

## Abstract

Conventional low-magnification phase-contrast microscopy is an invaluable, yet a qualitative, imaging tool for the interrogation of transparent objects over a mesoscopic millimeter-scale field-of-view in physical and biological settings. Here, we demonstrate that introducing a compact, unbalanced phase-shifting Michelson interferometer into a standard reflected brightfield microscope equipped with low-power infinity-corrected objectives and white light illumination forms a phase mesoscope that retrieves remotely and quantitatively the reflection phase distribution of thin, transparent, and weakly scattering samples with high temporal (1.38 nm) and spatial (0.87 nm) axial-displacement sensitivity and micrometer lateral resolution (2.3 μm) across a mesoscopic field-of-view (2.25 × 1.19 mm^2^). Using the system, we evaluate the etch-depth uniformity of a large-area nanometer-thick glass grating and show quantitative mesoscopic maps of the optical thickness of human cancer cells without any area scanning. Furthermore, we provide proof-of-principle of the utility of the system for the quantitative monitoring of fluid dynamics within a wide region.

Low-magnification Zernike phase-contrast and Nomarski differential interference contrast (DIC) microscopes are the most traditionally employed light systems for the non-destructive label-free visualization of optically transparent objects over a mesoscopic field-of-view (FOV) at micrometer lateral resolution. Despite the fact that these classical methods offer a wide range of applications in large-area optical phase imaging, including, for example, interrogation of biological cells and materials^1^,^2^,^3^,^4^,^5^, Zernike and DIC microscopes only provide qualitative information about the phase of the sample under observation. Furthermore, the inherent nonlinearly coupled phase-amplitude contrast mechanism governing the operation of these microscopes yields artifacts that make quantitative processing and analysis of imaging data across a large-area difficult. To facilitate these tasks, an accurate linear mapping of minute specimen-induced phase variations is therefore highly desirable.

During the past decade, a multitude of light microscopy methods has been devised to quantitatively measure optical phase shifts (associated with changes in the optical thickness of the sample) by taking advantage of optical interferometry and digital holography. While the underlying performance and principles of operation vary among the diverse methods, the majority of the techniques have been optimized for medium to high magnification observation, which is adequate for quantitative phase imaging over a relatively small FOV (<0.5 mm^2^)^6^,^7^,^8^,^9^,^10^,^11^,^12^,^13^,^14^,^15^,^16^,^17^,^18^,^19^. However, for quantitative optical phase measurements across a mesoscopic region (≥1 mm^2^), low magnification observation using low numerical-aperture (NA) imaging optics is preferred. Along this line, full-field swept-source phase microscopy has been devised in 2006 to nano-profiling the surface of a millimeter-sized printed DNA array in ~60 s^20^. A different approach for wide-field quantitative phase imaging based on near-common-path off-axis interferometry has lately been demonstrated to visualize the topography of millimeter-sized reflective and transmissive samples with nanometer axial-displacement sensitivity across impressively large FOVs of up to ~232 mm^2^, but with lateral resolution of several tens of micrometers^21^,^22^. Recently, Rinehart *et al.* have reported an off-axis Mach-Zehnder interferometric system that utilizes low magnification objectives to provide quantitative phase transmission imaging of water-soluble polymeric films with high temporal axial-displacement sensitivity (1.14 nm) and micrometer resolution (6.4 μm) across a millimeter-scale FOV (2 × 1.5 mm^2^)^23^. In particular, Rinehart *et al.* have demonstrated the effectiveness of their instrument in an important application involving the evaluation of microbicide films for anti-HIV drug delivery. However, the system employs a highly coherent illumination source, and thus can suffer from parasitic interferences and coherent noise that affect the spatial axial-displacement sensitivity^24^. Another method of wide-field quantitative phase imaging using low-NA microscope objectives is Fourier ptychographic microscopy^25^,^26^, which has a robust ability to reconstruct quantitative high-resolution phase maps of thin samples across a notably large area of ~120 mm^2^. However, Fourier ptychography requires the acquisition of a numerous number of images over several minutes and is computationally expensive. Extensions of Fourier ptychography to phase imaging at faster data acquisition rates and phase imaging in 3D have been lately realized^27^,^28^; yet, the question how well the phase acquired through these new developments matches quantitatively the actual phase profile of the sample remains to be investigated. Importantly, in recent years, novel lensless on-chip imaging modalities based on digital inline holography have been developed to obtain phase-contrast imaging with high sub-micron resolution over a wide FOV (~24–30 mm^2^)^29^,^30^. Although these modalities provide better visualization of transparent, weakly scattering objects with simple instrumentation (but involved mathematical processing), it still remains to assess their stability against phase noise, and quantify their measurement sensitivity to minute optical path-length changes.

In this work, we introduce a technique termed quantitative phase mesoscopy (QPMES) that enables the visualization of the reflection phase distribution of thin, label-free, optically transparent and weakly scattering specimens at micrometer resolution over a mesoscopic FOV with no area scanning and with nanometer spatiotemporal sensitivity to optical path-length changes. The term mesoscopy conveys the ability of the method to directly image objects at micrometer lateral resolution across a millimeter-scale area. The technique employs a low-NA, low-coherence tandem interferometric (LCTI) arrangement. Specifically, LCTI includes a wide, spatially incoherent and temporally low-coherent beam (from a white light source) that illuminates a sensing interferometer, which consists of a thin, unlabeled, transparent and weakly scattering sample placed on a glass coverslip, and a receiving interferometer comprising a single compact Michelson interferometer. The measurement field reflected from the surface of the sample is combined, at the output of the sensing interferometer, with the field transmitted through the sample and back-reflected from the bottom surface of the coverslip (termed the reflector field). These two fields are paraxial (due to the low-NA optics employed) and generate a self-referencing interference pattern that conveys information about the optical thickness of the sample (or surface topography, if the refractive index of the sample is known). The high sensitivity phase contrast visualization of QPMES is accomplished through coherence tuning and phase shifting of these two fields by the receiving interferometer as presented in the Methods section and Supplementary Note 1. It is noteworthy that coherence tuning of temporally low-coherent light waves was originally demonstrated for multiplex optical communication and high-accuracy fiber-optic sensing^31^, and was also employed by Fang-Yenin *et al.* for noncontact point-measurements of nerve displacement during action potential^32^. We note that unlike the Michelson interferometer in^32^ (used only for coherence tuning at a single lateral point on the sample), the Michelson interferometer in this work is used for both coherence tuning and quantitative phase retrieval with high spatial and temporal phase sensitivity over a wide field-of-view with no area scanning, eliminating the need to use involved methods of high phase stability, such as phase-referenced interferometry^32^.

In contrast to single-transmission-mode quantitative phase imaging methods where the measured phase shifts are proportional to the refractive index difference between the sample and the surrounding medium Δ*n*, the phase probed by QPMES is relative to the mean refractive index of the sample, 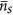, providing a 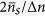-fold improvement in the axial-displacement sensitivity over single-transmission mode techniques for identical measurement phase noise levels^9^,^10^,^11^,^12^,^20^. Assuming shot noise limited detection, comparable phase noise levels can be obtained when the intensity detected from the sample is similar in both methods (e.g., when imaging semi-reflective materials). Otherwise, the much higher intensity detected from the sample in single-transmission-mode systems (e.g., when imaging biological cells) neutralizes the advantage of reflection-mode schemes in measuring accurate phase proportional to the sample refractive index at lower signal-to-noise ratios, consequently yielding similar phase sensitivity for both techniques as detailed in Supplementary Note 2.

Notably, unlike large-area phase imaging based on off-axis Mach-Zehnder interferometric systems and custom-built lensless on-chip digital inline holographic modalities, QPMES provides a novel and simple means for retrofitting most existing reflected brightfield microscopes into quantitative phase imagers with no need for area scanning using a single compact Michelson interferometer. Although this retrofitting can be realized in various topologies, here we incorporate a near-common-path phase-shifting Michelson interferometer into the infinity-corrected path of a home-built brightfield microscope equipped with white light epi-illumination and low power objectives. Note that the use of white light illumination contributes significantly to the high spatial sensitivity of QPMES, presenting a fundamental advantage over quantitative phase mesoscopes using laser light. We demonstrate that this system lends itself to subsequent image processing and analysis, and thus offers the basis for non-destructive direct examination of large-area etching topography at the nanoscale and quantitative mesoscopic imaging of the optical thickness of unlabeled biological cells without any area scanning. Furthermore, we provide proof-of-principle of the utility of the system for the quantitative monitoring of fluid dynamics within a large area.

## Methods

**Figure 1:**
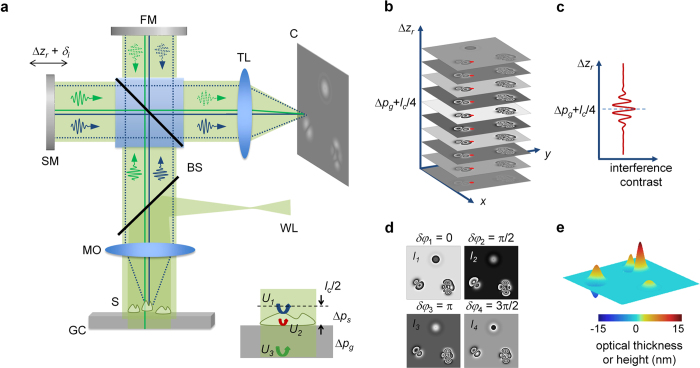
Quantitative reflection phase mesoscopy (QPMES). **(a)** Schematics of the QPMES system: WL, white light source; S, specimen; GC, glass coverslip; MO, 5×/0.14 infinity-corrected microscope objective; BS, 50/50 beam splitter; FM, fixed mirror of the receiving interferometer; SM, scanning mirror of the receiving interferometer translated by a nanopositioner; TL, tube lens; C, camera. The zoom inset shows the sensing interferometer where *U*_*1*_, *U*_*2*_ and *U*_*3*_ are the fields reflected from the sample surface (the measurement field; marked blue), sample-glass interface (marked red) and bottom surface of the coverslip (the reflector field; marked green), respectively. **(b,c)** Remote coherence tuning in QPMES. **(b)** In general, the OPD of the receiving interferometer is set to 2Δ*z*_*r*_ = 2Δ*p*_*g*_ + *l*_c_/2 by translating the scanning mirror, thereby positioning the center of the temporal coherence gate of the light source, with a roundtrip coherence length of *l*_c_/2, a distance of *l*_c_/4 above the sample-glass. **(c)** This position is determined by first maximizing the interferogram contrast at sample-free locations (i.e., 2Δ*z*_*r*_ = 2Δ*p*_*g*_) followed by an additional shift of the scanning mirror by *l*_c_/4 to reduce interferences from the sample-glass interface (red point shown in **(b)**). **(d,e)** Phase-shifting interferometry in QPMES. **(d)** Four phase-shifted interferograms are recorded and **(e)** processed using Equation (2) to retrieve a quantitative phase image of a thin sample (Δ*p*_*s*_ < *l*_c_/2) over an optical distance of one half of the source coherence length, *l*_c_, from the top surface of the coverslip as shown in the zoom inset of **(a)**. The image is subsequently unwrapped and converted to either an optical thickness image or a height (surface topography) image.

QPMES employs the principle of low-coherence tandem interferometry (LCTI)^31^,^32^, whose experimental arrangement is shown schematically in Fig. 1a. Light from a collimated white light source (KL 2500 LCD, Zeiss with a ET525/50m filter, Chroma) with a coherence length, *l*_c_, of ~5 μm in air is projected through a relay lens and a low magnification infinity-corrected objective to the sample plane (Mitutoyo Plan Apo Long WD 5×/0.14; 2.3-μm diffraction-limited resolution). As a result, the sample and the underlying microscope cover glass (#1.5 glass coverslip, Menzel) are uniformly illuminated over a wide FOV with ~0.25 mW/cm^2^. Assuming a <*l*_c_/2-optical thick, transparent and weakly scattering sample measured in air, spatially incoherent local paraxial reflections from the sample surface (*U*_*1*_), sample-glass interface (*U*_*2*_) and bottom surface of the coverslip (*U*_*3*_) are then naturally emerging out from the sensing interferometer as illustrated in the zoom inset of Fig. 1a. Note that for multiply scattering samples, the sample and underlying glass coverslip could be illuminated from below to produce a reference field unperturbed by scattering using reflections off the bottom surface of the coverslip. No interference pattern is virtually casted by the fields reflected from the sample surface (or the sample-glass interface) and the bottom surface of the coverslip as their optical path-length difference (OPD) is several tenfold larger than the coherence length of the illumination source. Moreover, the interference between the sample surface and sample-glass interface fields suffers from a very low signal-to-noise ratio, and is thus undetectable. As a result, there is no access to phase information about the sample. To enable the recovery of the sample phase distribution, the output of the sensing interferometer is coupled through the same objective into a compact unbalanced phase-shifting Michelson interferometer, termed the receiving interferometer. The scanning mirror of the receiving interferometer is translated, for instance, by a nanopositioner (Nano-OP30, Mad City Labs). The receiving interferometer output is then projected by a tube lens (*f* = 200 mm or *f* = 300 mm, Achromatic Doublets, Thorlabs) onto the camera (Lt225, Lumenera), which acquires phase-shifted interferograms (with 8-bit depth and integration time of 31–48 ms using LabVIEW 2014, National Instruments).

The spatial irradiance recorded reads as^31^,^32^


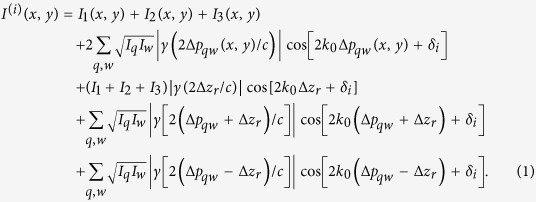


Here, (*q*, *w*) = {(1, 2), (1, 3), (2, 3)} are the indices of the interfering fields *U*_*q*_ and *U*_*w*_ where *U*_*1*_, *U*_*2*_, *U*_*3*_ denote the fields reflected from the sample surface (measurement field), sample-glass interface and bottom surface of the coverslip (reflector field), respectively, as depicted in the zoom inset of Fig. 1a. Also, *I*_*1*_, *I*_*2*_ and *I*_*3*_ are the mean intensity distributions of the fields *U*_*1*_, *U*_*2*_, *U*_*3*_, respectively, γ (.) is the normalized autocorrelation function of the light source, *c* is the speed of light in air and *k*_0_ is the source center wavenumber. Lastly, 2Δ*z*_*r*_ represents the OPD of the receiving interferometer, δ_*i*_ = 0, π, π/2, 3π/2; *i* = 1, 2, 3, 4 symbolize the discrete phase shifts introduced by the receiving interferometer, and 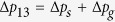, 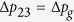, and 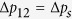 with Δ*p*_*g*_ and Δ*p*_*s*_*(x, y)* being the optical thickness of the glass coverslip and sample, respectively. Note that 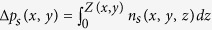 where 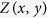 and 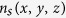 are the surface topography and refractive index of the sample, respectively.

By inspecting Equation (1) and recognizing that 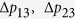 ≫ *l*_c_/2 (since the optical thickness of the glass coverslip is several tenfold larger than the illumination coherence length) and that the interference pattern generated by the sample surface and sample-glass interface fields is masked by noise, we see that the terms of the second and fourth rows in Equation (1) vanish. In addition, when the OPD of the receiving interferometer, 2Δ*z*_*r*_, is set to be much larger than the coherence length of the source (Δ*z*_*r*_ ≫ *l*_c_/2) the terms of the third row in Equation (1) can be eliminated. Setting the OPD of the receiving interferometer to 2Δ*z*_*r*_ = 2Δ*p*_*g*_ + *l*_c_/2 (thus positioning the center of the temporal coherence gate a distance of *l*_c_/4 above the sample-glass interface as shown in Fig. 1a–c) and assuming a <*l*_c_/2-optical thick weakly scattering sample, quadrant phase shift interference patterns are produced between the measurement and reflector fields (i.e., *U*_*1*_ and *U*_*3*_) and between the field reflected from the sample-glass interface and the reflector field (i.e., *U*_*2*_ and *U*_*3*_). These interference signals correspond to terms (*q,w*) = {(1,3), (2,3)} in the fifth row of Equation (1), respectively. Note that the term corresponding to (*q,w*) = {(1,2)} in the fifth row of Equation (1) vanishes because |Δ*p*_*s*_−Δ*z*_*r*_ | ≫ *l*_c_/2. To retrieve a quantitative phase image of the sample over an optical distance of *l*_c_/2 from the top surface of the coverslip (zoom inset of Fig. 1a), four phase-shifted interferogrmas, 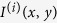, (Fig. 1d) are recorded and processed using the following relationship as detailed in Supplementary Note 1^33^


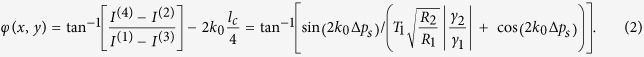


Here, 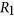 and 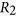 represent reflectivities from the sample surface and the sample-glass interface and 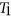 is the transmissivity of the sample surface. Also, 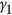 and 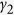 are the degrees of temporal coherence of the illumination source evaluated at 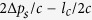 and 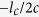, respectively. As observed from Equation (2), the resulting phase image *φ(x, y)* in QPMES is quantitative relative to the phase of the top surface of the coverslip at sample-free locations. The phase image is subsequently unwrapped using the Goldstein’s branch cut algorithm^34^ and converted into a topographic height image when the refractive index of the sample is known. Otherwise, the sample’s optical thickness image is presented (Fig. 1e).

Importantly, Equation (2) indicates that for samples arranged to be imaged on a glass slide in air the actual optical thickness of the sample, 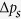, can be retrieved by QPMES when 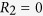 or 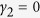. Otherwise, the nonlinearity of the inverse tangent induces deviations of *φ(x, y)* from 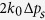 as detailed in Supplementary Note 1. In general, we note that these deviations are minimized when *R*_*2*_ ≪ *R*_*1*_ and/or when the temporal coherence gate filters out signals reflected off the sample-glass interface (for instance, by shifting the coherence gate further away from the top surface of the coverslip). Furthermore, we note that Equation (2) can also be used to retrieve the phase of the QPMES image when the glass coverslip and the sample are illuminated from below. Finally, Equation (2) with 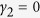 describes the QPMES phase image in other sample arrangements (e.g., similar to those used in Refs. 9, 11, 12) for which the receiving interferometer is tuned to accurately recover the total/depth-resolved optical thickness of the sample from the interference of the measurement and reflector fields while suppressing unwanted interferences from other interfaces (owing to the coherence gating inherent to QPMES).

## Results and Discussion

**Figure 2:**
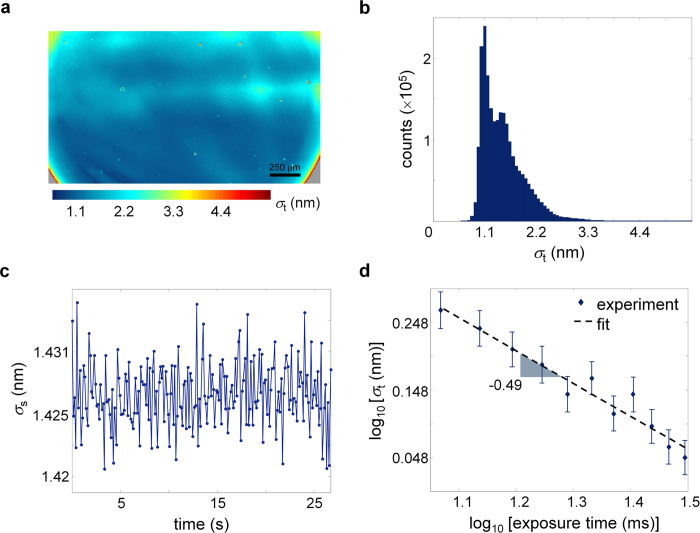
Characterization of the axial-displacement sensitivity of the QPMES prototype. The image set used for the sensitivity analysis included 200 topographic height images of the top surface of a cleaned unprocessed ~170 mm-thick glass coverslip acquired over a FOV of 2.25 × 1.19 mm^2^ with 2.3 μm lateral resolution at a rate of 7.98 height-images per second. **(a)** Spatial map of the temporal variation of the height, σ_t_(*x*, *y*), across the entire FOV. **(b)** Histogram of σ_t_(*x*, *y*). **(c**) Dependence of the spatial standard deviation of the height image on time, σ_s_(*t*). **(d)** Power relationship between the measured temporal axial-displacement sensitivity and the camera exposure time.

To characterize the temporal and spatial sensitivity levels of the QPMES system, we recorded 800 phase-shifted interferograms of a cleaned unprocessed glass coverslip of ~170 μm thickness with a 5× objective at 31.94 interferograms per second over 25.05 s. The NA of the objective was 0.14, which provides a diffraction limited lateral resolution of 2.3 μm. From these interferograms, a set of 200 topographic height images of the top surface of the coverslip was recovered following curvature removal across a FOV of 2.25 × 1.19 mm^2^, limited only by the camera sensor size (2048 × 1088 pixels, 5.5 × 5.5 μm^2^ pixel size). Note that 5× objectives with higher-NA could improve the lateral resolution of the QPMES prototype (down to ~1.4 μm), whereas lower-NA objectives (down to ~0.03) could significantly increase the imaging FOV of the system, but at the expense of lower lateral resolution (up to 10 μm). The temporal standard deviation of the height, σ_t_, was computed for each point in the image set to yield the spatial map of the standard deviation, σ_t_(*x, y*), over the entire FOV (Fig. 2a). The median value of the histogram of σ_t_(*x, y*) (Fig. 2b) was extracted to be 1.38 nm and it determines the temporal axial-displacement sensitivity of the QPMES instrument. Similarly, we computed the dependence of the spatial standard deviation of the height image on time, σ_s_(*t*), and obtained a median value of 1.43 nm (Fig. 2c). Note that this value represents the root-mean-square roughness of the unprocessed coverslip surface rather than the spatial axial-displacement sensitivity of the QPMES setup as described later in the text. Nevertheless, the measured values of σ_t_ and σ_s_ indicate the high spatiotemporal phase stability of the instrument.

QPMES imaging in the shot noise temporal sensitivity limit is critical in order to obtain the minimum achievable temporal phase noise. Theoretical predictions relate the temporal phase noise floor of a shot noise limited system to the square root of the reciprocal of the camera exposure time^9^,^10^,^20^ and Supplementary Note 2]. We therefore measured the temporal axial-displacement sensitivity of the system as a function of the camera integration time using topographic height images of an unprocessed glass coverslide. The images were generated as described above, and for each exposure time, the temporal sensitivity was evaluated as before. Figure 2d presents the dependence of the temporal axial-displacement sensitivity on the exposure time in double logarithmic scale. The slope of the linear fit to the experimental data is −0.49, compatible with the theoretical predictions. Accordingly, this result validates the operation of the QPMES system in the shot noise temporal sensitivity limit.

The nanometer temporal sensitivity and surface roughness measured over a millimeter-scale FOV with micrometer lateral resolution suggest that QPMES would be an excellent starting point for scanningless quantitative mesoscopic phase imaging and analysis of physical and biological systems. As exemplary demonstrations in critical application fields of large-area optical phase imaging, we used the QPMES prototype for scan-free quantitative examination of a glass-etched grating structure and fixed unlabeled biological cells across a mesoscopic FOV. In addition, we provide proof-of-principle of the utility of the system for scan-free quantitative monitoring of fluid dynamics within a wide area.

**Figure 3:**
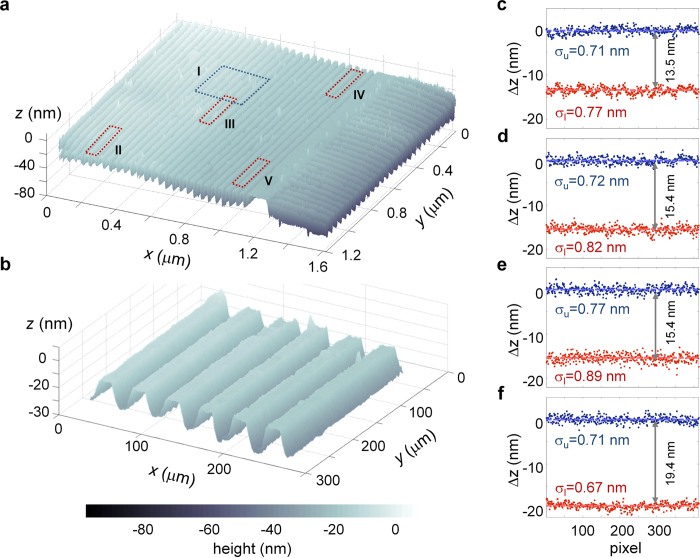
Direct non-destructive mesoscopic phase inspection of large-area etching topography at the nanoscale by QPMES. **(a)** Topographic height image of a glass-etched grating structure across a FOV of 1.6 × 1.19 mm^2^. **(b)** Zoom image of region I marked blue in **(a)**. **(c–f)** Automated scatter display of height measurements at the upper and lower surface of the grating grooves along their resolved orientation in regions **(c)** II, **(d)** III, **(e)** IV and **(f)** V marked red in **(a)**.

At first, we imaged a fabricated grating phase mask comprising vertically and horizontally oriented grooves etched on a glass coverslip of ~170 μm thickness. These grooves exhibited depth levels of ~13–17 nm across the mask as measured by an atomic force microscope (AFM). Note that here the OPD of the receiving interferometer was set to 2Δ*z*_*r*_ = 2Δ*p*_*g*_ in order to place the top surface of the coverslip within the temporal coherence gate. Also, it is necessary to point out that the QPMES image phase for this structure was obtained from Equation (2) with *R*_*2*_ = 0, thus recovering the actual surface topography of the sample. For scanningless imaging of the optical thickness of the sample, four phase-shifted interferograms were acquired with a 5× objective within a total time of 0.125 s by the QPMES system. The interferograms were then processed according to Equation (2) and unwrapped to yield the optical thickness image following curvature removal. The optical thickness image was subsequently converted to a height image by dividing the optical thickness by the refractive index of the glass coverslip (*n*_*g*_ = 1.51), resulting in a three-dimensional (3D) etch-depth map across a mesoscopic area of 1.6 × 1.19 mm^2^ (Fig. 3a). The etch-depth image zooming in on the details of an arbitrarily selected 300 × 300 μm^2^ area in Fig. 3a (marked blue) shows grooves of 16.4 nm median depth (Fig. 3b), which is compatible with the AFM observation of the grooves.

The ability to measure precisely etch-depth over a wide area is essential for the automated quantification of etch-depth uniformity in multiple locations across a large sample. This task remains an important need particularly in high-volume manufacturing processes. To this end, we applied to the QPMES topographic height data of Fig. 3a a computerized algorithm that determines the groove direction in selected areas across the sample (via Fourier analysis^35^) and presents the scatter of the height measurements in the upper and lower surface of the grooves along their resolved orientation (Fig. 3c–f). Median values of 13.5–19.4 nm for the groove etch-depth and 0.67–0.89 nm for the groove root-mean-square surface roughness were extracted from this scatter data. These values reveal subtle yet pertinent information regarding the quality of the etching process and the spatial sensitivity of the QPMES instrument. In particular, the min-max etch-depth non-uniformity of the process was found to be 17.9%, indicating the relatively low quality of the fabricated grating phase mask. In addition, the ability of the QPMES instrument to measure the smoother surface roughness of the grating groove structure—more than 1.6-fold smaller than the median roughness of the unprocessed glass coverslip (due to the etching process)—implies on the remarkably high spatial axial-displacement sensitivity of the system. Indeed, we obtained median values of 0.87 nm and 1.07 nm for the standard deviation of the height of the upper and lower surface of the grooves, respectively, using two hundred topographic images of the manufactured grating mask over a FOV of 1.6 × 1.19 mm^2^, resulting in a spatial sensitivity of 0.87 nm for the current QPMES prototype. Note that the use of illumination from a standard white light source contributed significantly to this high level of spatial sensitivity, presenting a fundamental advantage over quantitative phase mesoscopes using laser light.

**Figure 4:**
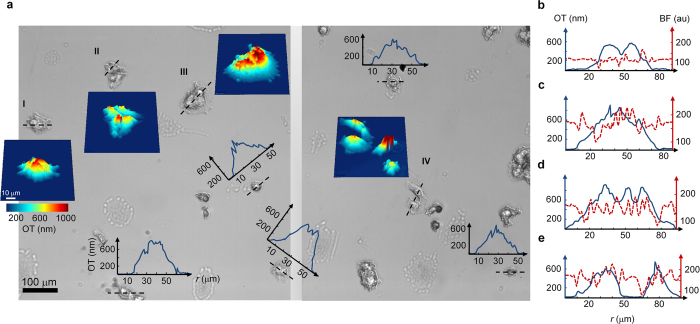
Scan-free quantitative mesoscopic phase imaging of unlabeled, fixed, dried MCF-7 cells in air by QPMES. **(a)** Part of the FOV of two 1.5 × 0.8 mm^2^ brightfield images from different regions of the sample together with several profiles of the optical thickness along the dashed line (passing through the cell nuclei) and 3D topographic optical thickness maps. Brightfield images were obtained by averaging the quadrature phase-shifted interferogram data. **(b–e)** Comparison of QPMES and brightfield signal contrasts. Optical thickness (OT, solid blue) and brightfield (BF, dashed red) line profiles through the cell nuclei are shown for regions **(b)** I, **(c)** II, **(d)** III and **(e)** IV displayed in **(a)**.

Another demonstration of QPMES includes the quantitative mesoscopic imaging of unlabeled human breast cancer MCF-7 cells. For imaging, cells were grown on a ~170 μm-thick glass coverslip for 48 hours and fixed in 3% paraformaldehyde. QPMES observation was performed by collecting a phase image of the fixed, dried cells in air in order to maximize the reflectivity from the cell surface and minimize the cell-glass refractive index mismatch. The optical thickness of the cells was thinner than *l*_*c*_/2, enabling measurement of their total optical thickness. The phase image was acquired within 0.125 s using a 5× objective and without the need for any objective lens, illumination beam or sample scanning. The phase image was then unwrapped to obtain the final image of the optical thickness of the cells. No curvature removal was required here (due to the sparse distribution of the cell colony) and a total magnification of 7.5× was implemented, yielding an imaging FOV of 1.5 × 0.8 mm^2^. Note that the acquisition time of the phase image could be considerably improved, while preserving similar spatiotemporal axial-displacement sensitivity, by using brighter illumination. Figure 4a shows two truncated brightfield images of the MCF-7 cells from different 1.5 × 0.8 mm^2^ regions of the sample alongside with several line profiles and 3D topographic maps of the cell optical thickness. While it is difficult to interpret information about the cell morphology from the brightfield images, the QPMES data effectively reveals morphological details of the cells including for example the optically thick cell nuclei (marked red in the 3D topographic maps in Fig. 4a). In this context, it is worth mentioning that similar quantitative topographic phase image data of biological cells measured in air was provided by spectral-domain phase microscopy^20^. To further illustrate the quantitative nature of QPMES versus the qualitative character of brightfield microscopy, we compared the signal contrast in the QPMES and brightfield images by extracting line profiles through the cell nuclei from images of the same field (Fig. 4b–e). The presence of multiple cell nuclei is apparent from the discernible peaks in the QPMES optical thickness profiles, however, multiple cell nuclei cannot be observed directly in the brightfield profiles. The reason for that is the ability of brightfield microscopy to produce high contrast only at the cell edges, making difficult the interpretation of morphological cell data.

**Figure 5:**
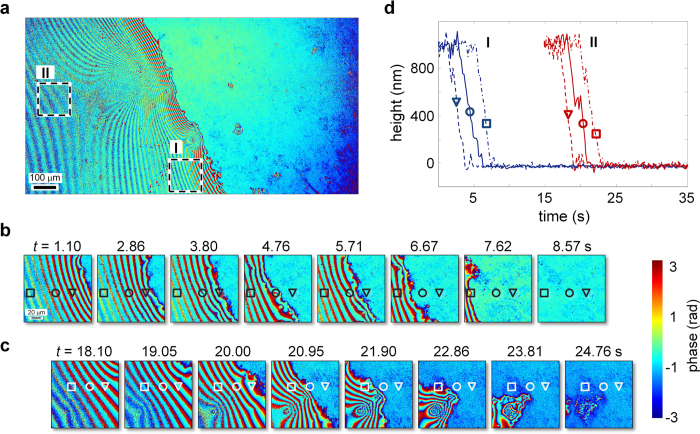
Direct quantitative mesoscopic phase monitoring of the dynamics of a water droplet on glass by QPMES. **(a)** Wrapped phase image of a water droplet over a wide FOV of 1.5×0.8 mm^2^ at *t* = 0 s. **(b,c)** Wrapped phase snapshots of the water droplet at selected instants during the course of the droplet dynamics across regions **(b)** I and **(c)** II marked with dashed line in **(a)**. **(d)** Temporal height profiles of the water droplet at different locations symbolized by ?, ? and ∇ in regions I and II shown in **(b)** and **(c)**, respectively. These height profiles were computed by unwrapping the phase image data using the Goldstein’s branch cut algorithm while referencing all time frames to a single identical region that remained free of water over the entire course of the experiment.

In addition to facilitating scan-free quantitative phase mesoscopy and nanoscale image analysis in material etching and cell imaging applications, QPMES can enable fluid dynamics to be monitored quantitatively in a wide FOV at micrometer lateral resolution without any area scanning. In a proof-of-concept demonstration, we imaged the dynamics of a water droplet on a ~170 μm-thick glass coverslip at room temperature over a mesoscopic FOV using the QPMES setup with a 0.14-NA objective and a magnification of 7.5× at 5.25 phase-images per second over 35 s. Although the phase-image acquisition rate of the current instrument is slower than that obtained by off-axis interferometric systems^18^,^23^, it could be improved up to tenfold by using brighter illumination and a three-step phase shifting algorithm. A 1.5 × 0.8 mm^2^ wrapped phase snapshot of the droplet at *t* = 0 s is shown in Fig. 5a, where selected time snapshots of the wrapped droplet phase across two regions in Fig. 5a appear in Fig. 5b,c. A close examination of the wrapped time-snapshots depicted in Fig. 5a–c unravels image sections of low phase contrast, a result of a long droplet height that exceeded the coherence length of the light source (left of Fig. 5a, bottom left of Fig. 5b,c at *t* = 1.10, 2.86, 3.80 s and *t* = 18.10, 19.05, 20.00 s, respectively). By unwrapping each time frame of the phase data set using the Goldstein’s branch cut algorithm^34^ while referencing all time frames to a single identical region that was water-free over the entire course of the experiment, the dynamics of the droplet height across regions I and II (outlined in Fig. 5a by a dashed line) was quantified as shown in Fig. 5d and in Supplementary Movie 1. The temporal evolution of the droplet height clearly reveals a second-scale dynamics at the different positions across the coverslip (Fig. 5d), confirming that a 5.25 Hz sampling rate is sufficient for adequately probing relatively slow dynamical processes with sub-second time resolution.

## Conclusions

We have presented quantitative phase mesoscopy (QPMES) that employs remote coherence tuning of phase-shift interference patterns to enable mapping of the reflection phase distribution of thin, label-free, optically transparent, and weakly scattering samples with micrometer lateral resolution and nanometer spatiotemporal axial-displacement sensitivity across a mesoscopic (millimeter-scale) area. Significantly, QPMES offers the possibility for converting most existing reflected light microscopes equipped with white light illumination and low power objectives into a quantitative phase mesoscope, for example, by incorporating into their infinity-corrected path a single compact Michelson interferometer.

We have demonstrated the usefulness of QPMES in critical application fields of large-area optical phase imaging including non-destructive direct inspection of etching topography at the nanoscale and scan-free quantitative visualization of the optical thickness of unlabeled biological cells. In the context of biological imaging, QPMES is expected to be useful for nano-profiling of structural changes in fixed cells/thin sections of tissue (due to abnormal growth, for example) and sensing of DNA biochips over a large area^20^. Extensions of QPMES to imaging of minute phase variations induced in living cells are possible, for instance, by tuning the coherence gate of the QPMES system to the interference between reflections from the surfaces of an imaging glass chamber within which cells are embedded^9^, or alternatively, tuning the system to the interference between the cell surface and the top surface of the chamber using sufficiently broadband illumination to enable depth-resolved phase information on the sample^11^,^12^. Both extensions require the use of a sufficiently bright light source to provide an adequately high signal-to-noise ratio of the interference pattern, and thus high axial-displacement sensitivity.

Finally, we provided proof-of-principle of the utility of QPMES for quantitative monitoring of fluid dynamics within a wide area at sub-second time resolution. At this temporal resolution, it should be possible to use the QPMES instrument either for quantitative phase measurements of biological and physical systems in time-lapse mode or for quantitative phase imaging of many relatively slow dynamical processes in physical and biological settings. For viewing fast dynamical processes with QPMES, a large megapixel high-speed camera together with brighter white light illumination could be employed in the current QPMES prototype. For example, commercially available four megapixel cameras with a full-resolution frame rate of 1450 frames per second (Phantom v641, Vision Research) would have provided QPMES with lateral resolution of ~4 μm across FOVs of ~16 mm^2^ at over 350 Hz.

## Additional Information

**How to cite this article**: Arbel, E. and Bilenca, A. Quantitative reflection phase mesoscopy by remote coherence tuning of phase-shift interference patterns. *Sci. Rep.* **5**, 12560; doi: 10.1038/srep12560 (2015).

## Supplementary Material

Supplementary Information

Supplementary Movie1

## References

[b1] Salvucci O. *et al.* Regulation of endothelial cell branching morphogenesis by endogenous chemokine stromal-derived factor-1. Blood 99, 2703–2711 (2002). 11929756 10.1182/blood.v99.8.2703

[b2] Hasegawa T. *et al.* Effect of fibroblast growth factor-2 on periodontal ligament cells derived from human deciduous teeth *in vitro*. Exp. Ther. Med. 1, 337–341 (2010). 22993547 10.3892/etm_00000052 PMC3445930

[b3] Oh C., Isikman S. O., Khademhosseinieh B. & Ozcan A. On-chip differential interference contrast microscopy using lensless digital holography. Opt. Express 18, 4717–4726, 10.1364/OE.18.004717 (2010). 20389485 PMC2859902

[b4] San Martín D., Palizdar Y., Cochrane R. C., Brydson R. & Scott A. J. Application of Nomarski differential interference contrast microscopy to highlight the prior austenite grain boundaries revealed by thermal etching. Mater. Charact. 61, 584–588 (2010).

[b5] Shah V. A. *et al.* Flat single crystal Ge membranes for sensors and opto-electronic integrated circuitry. Solid State Electron. 98, 93–98 (2014).

[b6] Yang C. *et al.* Phase-referenced interferometer with subwavelength and subhertz sensitivity applied to the study of cell membrane dynamics. Opt. Lett. 26, 1271–1273 (2001). 18049583 10.1364/ol.26.001271

[b7] Iwai H. *et al.* Quantitative phase imaging using actively stabilized phase-shifting low-coherence interferometry. Opt. Lett. 29, 2399–2401 (2004). 15532280 10.1364/ol.29.002399

[b8] Marquet P. *et al.* Digital holographic microscopy: a noninvasive contrast imaging technique allowing quantitative visualization of living cells with subwavelength axial accuracy. Opt. Lett. 30, 468–470 (2005). 15789705 10.1364/ol.30.000468

[b9] Joo C., Akkin T., Cense B., Park B. H. & De Boer J. F. Spectral-domain optical coherence phase microscopy for quantitative phase-contrast imaging. Opt. Lett. 30, 2131–2133 (2005). 16127933 10.1364/ol.30.002131

[b10] Choma M. A., Ellerbee A. K., Yang C., Creazzo T. L. & Izatt J. A. Spectral-domain phase microscopy. Opt. Lett. 30, 1162–1164 (2005). 15945141 10.1364/ol.30.001162

[b11] Yamauchi T., Iwai H., Miwa M. & Yamashita Y. Low-coherent quantitative phase microscope for nanometer-scale measurement of living cells morphology. Opt. Express 16, 12227–12238, 10.1364/OE.16.012227 (2008). 18679500

[b12] Yaqoob Z. *et al.* Single-shot full-field reflection phase microscopy. Opt. Express 19, 7587–7595, 10.1364/OE.19.007587 (2011). 21503067 PMC3368324

[b13] Shaked N. T. Quantitative phase microscopy of biological samples using a portable interferometer. Opt. Lett. 37, 2016–2018 (2012). 22660106 10.1364/OL.37.002016

[b14] Bhaduri B., Tangella K. & Popescu G. Fourier phase microscopy with white light. Biomed. Opt. Express 4, 1434–1441, 10.1364/BOE.4.001434 (2013). 24010005 PMC3756570

[b15] Lyulko O. V., Randers-Pehrson G. & Brenner D. J. Simultaneous immersion Mirau interferometry. Rev. Sci. Instrum. 84, 053701 (2013). 23742552 10.1063/1.4803181 PMC3656945

[b16] Edwards C., Bhaduri B., Griffin B. G., Goddard L. L. & Popescu G. Epi-illumination diffraction phase microscopy with white light. Opt. Lett. 39, 6162–6165 (2014). 25361304 10.1364/OL.39.006162

[b17] Rappaz B., Breton B., Shaffer E. & Turcatti G. Digital Holographic Microscopy: a quantitative label-free microscopy technique for phenotypic screening. Comb. Chem. High Throughput Screen. 17, 80–88 (2014). 24152227 10.2174/13862073113166660062 PMC3894694

[b18] Girshovitz P. & Shaked N. T. Doubling the field of view in off-axis low-coherence interferometric imaging. Light Sci. Appl. 3, e151, 10.1038/lsa.2014.32 (2014).

[b19] Singh A. K., Faridian A., Gao P., Pedrini G. & Osten W. Quantitative phase imaging using a deep UV LED source. Opt. Lett. 39, 3468–3471 (2014). 24978513 10.1364/OL.39.003468

[b20] Sarunic M. V., Weinberg S. & Izatt J. A. Full-field swept-source phase microscopy. Opt. Lett. 31, 1462–1464 (2006). 16642139 10.1364/ol.31.001462

[b21] Gabai H. & Shaked N. T. Dual-channel low-coherence interferometry and its application to quantitative phase imaging of fingerprints. Opt. Express 20, 26906–26912, 10.1364/OE.20.026906 (2012). 23187544

[b22] Rajshekhar G. *et al.* Nanoscale topography and spatial light modulator characterization using wide-field quantitative phase imaging. Opt. Express 22, 3432–3438, 10.1364/OE.22.003432 (2014). 24663633

[b23] Rinehart M., Grab S., Rohan L., Katz D. & Wax A. Analysis of vaginal microbicide film hydration kinetics by quantitative imaging refractometry. PLoS One 9, e95005, 10.1371/journal.pone.0095005 (2014). 24736376 PMC3988224

[b24] Martínez-León L., Pedrini G. & Osten W. Applications of short-coherence digital holography in microscopy. Appl. Opt. 44, 3977–3984 (2005). 16004043 10.1364/ao.44.003977

[b25] Zheng G., Horstmeyer R. & Yang C. Wide-field, high-resolution Fourier ptychographic microscopy. Nat. Photonics 7, 739–745 (2013). 25243016 10.1038/nphoton.2013.187 PMC4169052

[b26] Ou X., Horstmeyer R., Yang C. & Zheng G. Quantitative phase imaging via Fourier ptychographic microscopy. Opt. Lett. 38, 4845–4848 (2013). 24322147 10.1364/OL.38.004845 PMC4277232

[b27] Tian L., Li X., Ramchandran K. & Waller L. Multiplexed coded illumination for Fourier Ptychography with an LED array microscope. Biomed. Opt. Express. 5, 2376–2389, 10.1364/BOE.5.002376 (2014). 25071971 PMC4102371

[b28] Tian L. & Waller L. 3D intensity and phase imaging from light field measurements in an LED array microscope. Optica 2, 104–111, 10.1364/OPTICA.2.000104 (2015).

[b29] Bishara W. *et al.* Holographic pixel super-resolution in portable lensless on-chip microscopy using a fiber-optic array. Lab Chip 11, 1276–1279 (2011). 21365087 10.1039/c0lc00684j PMC3151573

[b30] Greenbaum A., Sikora U. & Ozcan A. Field-portable wide-field microscopy of dense samples using multi-height pixel super-resolution based lensfree imaging. Lab Chip 12, 1242–1245 (2012). 22334329 10.1039/c2lc21072j

[b31] Rao Y. J. & Jackson D. A. Recent progress in fibre optic low-coherence interferometry. Meas. Sci. Technol. 7, 981–999 (1996).

[b32] Fang-Yen C., Chu M. C., Seung H. S., Dasari R. R. & Feld M. S. Noncontact measurement of nerve displacement during action potential with a dual-beam low-coherence interferometer. Opt. Lett. 29, 2028–2030 (2004). 15455769 10.1364/ol.29.002028

[b33] Schreiber H. & Bruning J. H. [Phase shifting interferometry] Optical Shop Testing, Third Edition [Malacara D. (ed.)] [547–666] (John Wiley & Sons, Inc., New Jersey, 2007).

[b34] Ghiglia D. & Pritt M. Two dimensional phase unwrapping: theory, algorithms & software. (John Wiley & Sons, Inc., New Jersey, 1998).

[b35] Hollitt C. & Deeb A. S. Determining image orientation using the Hough and Fourier transforms. in Proc. IVCNZ 2012, 346–351 (Association for Computing Machinery, 2012). 10.1145/2425836.2425904.

