# Supplementary material for: Quantitative reflection phase mesoscopy by remote coherence tuning of phase-shift interference patterns
Source: Sci Rep. 2015 Jul 28;5:12560. doi: 10.1038/srep12560 (PMC4517165; doi:10.1038/srep12560)
Supplement: Supplementary Information [file srep12560-s1.pdf]

SUPPLEMENTARY INFORMATION

Quantitative reflection phase mesoscopy by remote coherence tuning of phase-shift interference patterns

Elad Arbel[1] and Alberto Bilenca[1,2*]

[1]Biomedical Engineering Department, Ben-Gurion University of the Negev, 1 Ben Gurion Blvd, Be’er-Sheva 84105, Israel

[2]Ilse Katz Institute for Nanoscale Science and Technology, Ben-Gurion University of the Negev, 1 Ben Gurion Blvd, Be’er-Sheva 84105, Israel

e-mail: eladarbel@hotmail.com, *bilenca@bgu.ac.il

Supplementary Note 1. QPMES signal formation

Consider a thin, transparent and weakly scattering sample with a transmission function $T(\boldsymbol{r}) = e^{ik\Delta p_s(\boldsymbol{r})}$, $\boldsymbol{r} = (x, y)$ placed on a glass coverslip in air and illuminated by a broadband spatially incoherent plane wave as shown in Figure S1a. Here, k is the wavenumber of light and $\Delta p_s(\boldsymbol{r}) = \int_0^{Z(\boldsymbol{r})} n_s(\boldsymbol{r}, z)\,dz$ is the optical thickness of the sample with $Z(\boldsymbol{r})$ and $n_s(\boldsymbol{r}, z)$ being its surface topography and refractive index, respectively. QPMES is aimed in this work at measuring $\Delta p_s(\boldsymbol{r})$ for $\Delta p_s(\boldsymbol{r}) < l_c/2$ where l_c denotes the coherence length of the illumination source.

To understand the formation of the QPMES signal, it is instructive to derive the interference signals at the output of the sensing interferometer for a completely spatially incoherent illumination field. Assuming low numerical aperture light illumination and collection (so that electric fields are paraxial), the interferometric components of these interference signals can be expressed as

$$\sum_{q,w} 2\,\mathrm{Re}\left\{\left\langle U_q(\boldsymbol{r}, t) U_w^*(\boldsymbol{r}, t + \tau_{qw})\right\rangle\right\} = \sum_{q,w} 2\sqrt{I_q(\boldsymbol{r}) I_w(\boldsymbol{r})} \left|\gamma\left(\frac{2\Delta p_{qw}(\boldsymbol{r})}{c}\right)\right| \cos\left[2k_0 \Delta p_{qw}(\boldsymbol{r})\right], \tag{1}$$

where $\mathrm{Re}\{X\}$ denotes the real part of the complex number *X* and $\langle \ \rangle$ represents time averaging due to the ergodicity (and hence stationarity) of the electric fields U_q and U_w. Here, $I_q(\boldsymbol{r})$ and $I_w(\boldsymbol{r})$ denote, respectively, the intensities of the fields U_q and U_w reflected from the surfaces indexed by *q* and *w* at the output of the sensing interferometer (Figure S1a), $\gamma(.)$ is the

normalized temporal autocorrelation function of the low-coherence illumination source and k_0 is the source center wavenumber. In addition, (q,w)={(1,2), (1,3), (2,3)} are the indices of the interfering fields $U_q(\boldsymbol{r},t)$ and $U_w(\boldsymbol{r},t+\tau_{qw})$ where $\tau_{qw}=\frac{2\Delta p_{qw}(\boldsymbol{r})}{c}$ is the time delay due to the difference in the length of the optical paths traversed by the fields with c being the speed of light in air. Specifically, $\Delta p_{12}=\Delta p_s$, $\Delta p_{13}=\Delta p_s+\Delta p_g$, $\Delta p_{23}=\Delta p_g$ where $\Delta p_g=n_g z_g$ with n_g and z_g denoting the refractive index and thickness of the glass coverslip, respectively.

For standard 170-μm thick glass coverslips and white light sources with a coherence length of l_c ~5 μm, Equation (1) suggests that retrieval of the total optical thickness of thin samples ($\Delta p_s < l_c/2$) involves coherence tuning of the measurement and reflector fields (U_1 and U_3, respectively) by an OPD of ~2×1.51×170+5/2=516 μm followed by introducing controllable phase differences between them. Note that the interference pattern produced by the field reflected from the sample surface, U_1, and the sample-glass-interface wave, U_2, suffers from a very low signal-to-noise ratio, and thus is undetectable. Also, it is noteworthy that QPMES could benefit from a strong signal reflected from the bottom surface of the coverslip (hence improving the detection signal-to-noise ratio), obtained, for instance, by coating the coverslip bottom surface with a highly reflective material. In general, U_1, U_2, U_3, the electric fields emerging out from the sensing interferometer, constitute the relevant fields for the formation of the QPMES signal. Following remote coherence tuning and temporal phase shifting by the receiving interferometer, the phase distribution associated with the QPMES image is relative to the phase of the top surface of the coverslip at sample-free locations. As illustrated in Figure S1b, the

QPMES image can be expressed as the phase of the complex sum of $\left\langle U_1(\boldsymbol{r},t)U_3^*\left(\boldsymbol{r},t+\frac{2\Delta p_s}{c}-\frac{l_c}{2c}\right)\right\rangle$ and $\left\langle U_2(\boldsymbol{r},t)U_3^*\left(\boldsymbol{r},t-\frac{l_c}{2c}\right)\right\rangle$

$$\begin{aligned}\varphi(\boldsymbol{r}) &= \measuredangle\left[\sqrt{I_1 I_3}\,|\gamma_1|\cos\left[2k_0\left(\Delta p_s - l_c/4\right)\right]+\sqrt{I_2 I_3}\,|\gamma_2|\cos\left[-2k_0\,l_c/4\right]\right]-2k_0\,\frac{l_c}{4} \\ &= \tan^{-1}\left[\sin\left(2k_0\Delta p_s\right)\Big/\left(T_1\sqrt{\frac{R_2}{R_1}}\left|\frac{\gamma_2}{\gamma_1}\right|+\cos\left(2k_0\Delta p_s\right)\right)\right],\end{aligned} \tag{2}$$

with R_1 and R_2 representing reflectivities from the sample surface and the sample-glass interface and T_1 being the transmissivity of the sample surface. Also, γ_1 and γ_2 are the degrees of temporal coherence of the illumination source evaluated at $2\Delta p_s/c - l_c/2c$ and $-l_c/2c$, respectively. Equation (2) indicates that for the sample arrangement used in this work (namely, samples placed on a glass slide and imaged in air) the actual optical thickness of the sample, Δp_s, can be retrieved by QPMES when $R_2 = 0$ or $\gamma_2 = 0$. Thus, deviations of $\varphi(\boldsymbol{r})$ from $2k_0\Delta p_s$ (due to nonlinearity of the inverse tangent) are minimized when $R_2 \ll R_1$ and/or when the temporal coherence gate filters out signals reflected off the sample-glass interface (for instance, by shifting the coherence gate further away from the top surface of the coverslip in the sample arrangement used here). Using Equation (2) with a Gaussian temporal coherence function with a full 1/*e* width of l_c/c and R_1, R_2, T_1 computed using Fresnel equations for low numerical aperture light illumination and collection, we can estimate the deviation of $\varphi(\boldsymbol{r})$ (from $2k_0\Delta p_s$) for the samples imaged throughout this work. Specifically, for the water droplet samples (assuming n_a=1, n_g=1.51, n_s=1.33 and Δp_s=200-1200 nm), Equation (2) predicts deviations on the order of 2.6-5.3% of 2π. Similarly, for the fixed dried MCF-7 cell samples (assuming n_a=1, n_g=1.51,

$\bar{n}_s \geq 1.39$ [ref. 11 in the main text] and Δp_s =200-1000 nm), Equation (2) predicts a maximum deviation of $\varphi(\mathbf{r})$ (from $2k_0\Delta p_s$) on the order of 1.6-3% of 2π. Note that the smaller deviations correspond to the larger values of Δp_s (due to the larger value of γ_1).

As a final remark, it should be pointed out that electric fields backscattered from weak optical heterogeneities inside the sample may occur in addition to the field reflected from the surface of the sample. Assuming low numerical aperture light illumination and collection, an equivalent sample-surface-reflected field, $\left\langle U_{1,eq}U_3^*\right\rangle$ (marked orange in Figure S1c), comprising the vector sum of the fields reflected and backscattered from the sample (marked solid and dashed blue in Figure S1c, respectively), is generated. Then, Equation (2) remains valid with $2k_0\Delta p_s$ and $T_1\sqrt{R_2}\,|\gamma_2| \big/ \sqrt{R_1}\,|\gamma_1|$ being substituted by the phase of $\left\langle U_{1,eq}U_3^*\right\rangle$ and the magnitude ratio of the vectors $\left\langle U_2U_3^*\right\rangle$ and $\left\langle U_{1,eq}U_3^*\right\rangle$, respectively, as illustrated in Figure S1c.

Supplementary Note 2. Axial-displacement sensitivity in reflection and single-transmission mode quantitative phase imaging.

Consider a specimen comprising a slab of mean refractive index $\bar{n}_s$ and thickness Δz placed in air, $n_a = 1$, at time $t = t_0$ (Figure S2a). A minute thickness of δz is then etched out from the slab at time $t = t_1$ as shown in the right panel of Figure S2a. To quantify δz, reflection and single-transmission mode quantitative phase imaging systems measure the phase difference between the time points $t = t_1$ and $t = t_0$, which reads as $\delta\varphi_R$ and $\delta\varphi_T$, respectively

$$\delta\varphi_R = \varphi_R\left(t_1\right) - \varphi_R\left(t_0\right) = 2k\bar{n}_s\left(\Delta z - \delta z\right) - 2k\bar{n}_s\Delta z = -2k\bar{n}_s\delta z \tag{3a}$$

$$\delta\varphi_T = \varphi_T\left(t_1\right) - \varphi_T\left(t_0\right) = \\ k\left(n_a\delta z + \bar{n}_s\left(\Delta z - \delta z\right)\right) + \phi_0 - \left(k\bar{n}_s\Delta z + \phi_0\right) = -k\left(\bar{n}_s - n_a\right)\delta z = -k\Delta n\delta z, \tag{3b}$$

where k is the wavenumber of light and Δn is the refractive index difference between the sample and surrounding medium (air in this example). Also, ϕ_0 represents the phase difference between the two arms of the single-transmission interferometer excluding the phase accumulated in the slab. Note that Equations (3a) and (3b) hold also for spatial phase measurements of a groove structure with a groove depth of δz. The root mean square noise for distance measurements (or axial-displacement sensitivity) of reflection and single-transmission mode quantitative phase imaging methods, δz_R^{sens} and δz_T^{sens}, respectively, can now be calculated to be

$$\delta z_R^{\text{sens}} = \sqrt{\left\langle \delta z^2 \right\rangle} = \frac{\sqrt{\left\langle \delta\varphi_R^2 \right\rangle}}{2k\bar{n}_s} \tag{4a}$$

$$\delta z_T^{\text{sens}} = \sqrt{\left\langle \delta z^2 \right\rangle} = \frac{\sqrt{\left\langle \delta\varphi_T^2 \right\rangle}}{k\Delta n}, \tag{4b}$$

where $\langle\ \rangle$ represents time/spatial averaging due to ergodicity of the measured optical phases. By inspecting Equations (4a) and (4b), we observe that for identical measurement phase noise levels, explicitly $\sqrt{\langle \delta\varphi_R^2 \rangle} = \sqrt{\langle \delta\varphi_T^2 \rangle}$, reflection-mode quantitative phase imaging provides a $2\bar{n}_s / \Delta n$-fold improvement in the axial-displacement sensitivity over single-transmission mode techniques.

To further compare the axial-displacement sensitivity of reflection and single-transmission mode quantitative phase imaging methods, $\sqrt{\langle \delta\varphi_R^2 \rangle}$ and $\sqrt{\langle \delta\varphi_T^2 \rangle}$ should be calculated. In general, $\sqrt{\langle \delta\varphi_R^2 \rangle}$ and $\sqrt{\langle \delta\varphi_T^2 \rangle}$ are limited by the signal-to-noise ratio (SNR) of the interference pattern. To understand this behavior, recall that the intensity of the interference pattern recorded in reflection and single-transmission mode quantitative phase imaging is given by

$$I_{\text{interference}} = I_{r,R/T} + I_{s,R/T} + 2\sqrt{I_{r,R/T} I_{s,R/T}} \cos\left(\varphi_{R/T}\right) + \mathrm{Re}\left\{ n_{\delta\varphi_{R/T}} \right\}, \tag{5}$$

with $I_{r,R/T}$ and $I_{s,R/T}$ being the intensities of the (self)reference and sample fields detected in reflection (R) and single-transmission (T) mode systems, respectively. Also, $\mathrm{Re}\{X\}$ denotes the real part of the complex number *X*, $\varphi_{R/T}$ is the phase difference between the two fields and $n_{\delta\varphi_{R/T}}$ represents the detection noise which is modeled by a temporarily and spatially zero-mean circularly-symmetric complex white Gaussian noise process with variance $\sigma^2 = 0.5 \cdot \left(I_{r,R/T} + I_{s,R/T} \right)$ (Figure S2b). Assuming that detection is limited by shot noise and that the phase noise is much weaker than $\varphi_{R/T}$, the root mean square phase noise in reflection and single-transmission mode quantitative phase imaging, $\sqrt{\langle \delta\varphi_{R/T}^2 \rangle}$, reads as

$$\sqrt{\left\langle \delta\varphi_{R/T}^2 \right\rangle} = \frac{C_{R/T}}{\sqrt{\mathrm{SNR}_{R/T}/2}}, \ \mathrm{SNR}_{R/T} = \frac{4I_{r,R/T}I_{s,R/T}}{I_{r,R/T}+I_{s,R/T}}, \tag{6}$$

with $C_{R/T}$ being a constant determined by the specific scheme of the reflection/single-transmission mode method. Combining Equation (6) and Equations (4a) and (4b) and assuming that $I_{r,R/T} \gg I_{s,R/T}$ or $I_{r,R/T} \approx I_{s,R/T}$, the ratio of the axial-displacement sensitivity of reflection and single-transmission mode phase imaging techniques can be expressed as

$$\frac{\delta z_T^{\mathrm{sens}}}{\delta z_R^{\mathrm{sens}}} = \frac{2\bar{n}_s}{\Delta n}\frac{C_T}{C_R}\sqrt{\frac{I_{s,R}}{I_{s,T}}}. \tag{7}$$

Although $C_{R/T}$, $I_{r,R/T}$, $I_{s,R/T}$ depend on the specific details of the reflection and single-transmission mode arrangement used, it is instructive to evaluate Equation (7) for the case where the only difference between the reflection and single-transmission mode systems stems from the intensity detected from the sample. Accordingly, substituting $C_R = C_T$, $I_{s,R} = R_s I_0$ and $I_{s,T} = \left(1-R_s\right)I_0$ into Equation (7) yields

$$\frac{\delta z_T^{\mathrm{sens}}}{\delta z_R^{\mathrm{sens}}} = \frac{2\bar{n}_s}{\Delta n}\sqrt{\frac{R_s}{1-R_s}}, \tag{8}$$

where I_0 is the total illumination intensity and $R_s \approx \left(\frac{\Delta n}{2\bar{n}_s - \Delta n}\right)^2$ for low numerical aperture light illumination and collection. Now, Equation (8) reveals that while $\delta z_R^{\mathrm{sens}} \approx \delta z_T^{\mathrm{sens}}$ for $R_s \to 0$ (for example, when imaging biological cells), $\delta z_R^{\mathrm{sens}} \approx {\Delta n}/{2\bar{n}_s}\, \delta z_T^{\mathrm{sens}}$ for $R_s \to 0.5$ (for example, when imaging semi-reflective materials). These results imply that in the shot noise detection limit, reflection-mode methods can offer a $\sim 2\bar{n}_s/\Delta n$-fold improvement in the axial-displacement sensitivity over single-transmission mode techniques once the intensity detected from the sample

is similar in both techniques; otherwise, the much higher intensity detected from the sample in single-transmission mode systems neutralizes the advantage of reflection-mode techniques in measuring accurate phase proportional to the sample refractive index at lower SNRs, thereby resulting in similar axial-displacement sensitivity for both reflection and single-transmission mode schemes.

Supplementary Figure S1. QPMES signal formation. **(a)** Electric fields at the output of the sensing interferometer. $U_1,\ U_2,\ U_3$ represent, respectively, the fields reflected from the sample surface located at $Z(\boldsymbol{r})$ (the measurement field), sample-glass interface at $z=0$ and glass-air interface at $z=-z_g$ (the reflector field). $n_a,\ n_s(\boldsymbol{r},z),\ n_g$ are the refractive indices of air, sample and glass, respectively, and $Z(\boldsymbol{r})$ is the surface topography of the sample. The center of the temporal coherence gate is located $l_c/4$ above the top surface of the coverslip, where l_c is the coherence length of the illumination source. Thick arrows indicate the direction of the incident light beam. **(b)** The QPMES image field (purple line) as the vector sum of $\left\langle U_1(\boldsymbol{r},t)U_3^*\left(\boldsymbol{r},t+\frac{2\Delta p_s}{c}-\frac{l_c}{2c}\right)\right\rangle$ (blue line) and $\left\langle U_2(\boldsymbol{r},t)U_3^*\left(\boldsymbol{r},t-\frac{l_c}{2c}\right)\right\rangle$ (red line) following remote coherence tuning and temporal phase shifting by the receiving interferometer. **(c)** The QPMES image field (purple line) for $\left\langle U_{1,eq}U_3^*\right\rangle$ (orange line) that includes fields reflected from the sample surface (solid blue line) and backscattered from weak optical heterogeneities inside the sample (dashed blue lines).

a

b

c

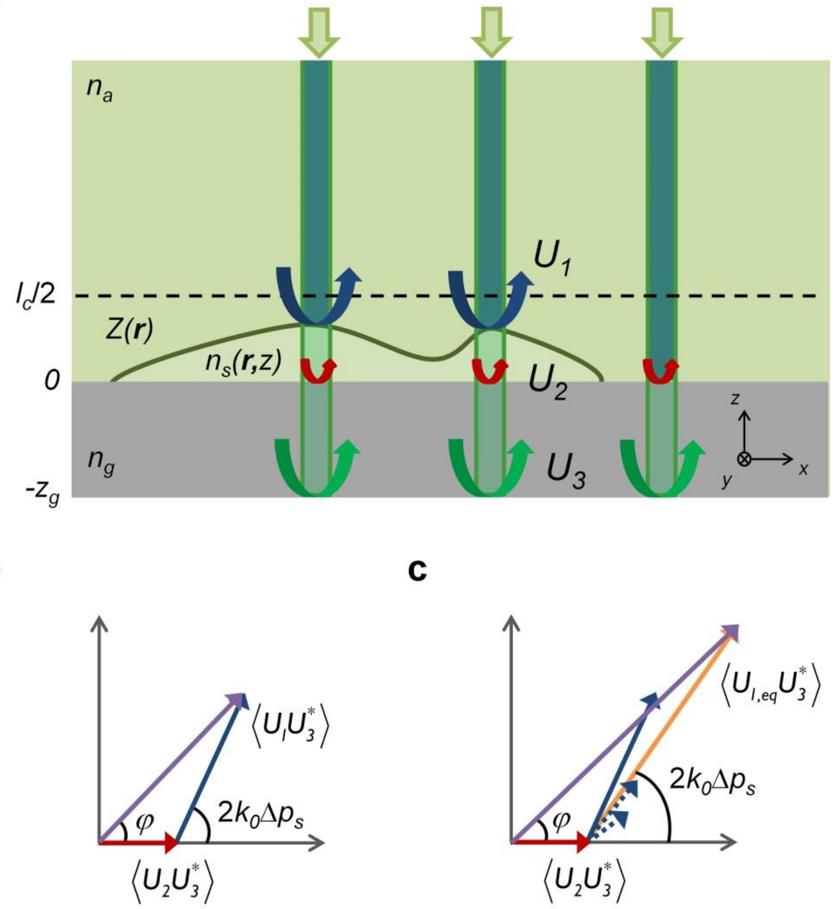

n_a
U_1
$l_c/2$
$Z(\mathbf{r})$
$n_s(\mathbf{r},z)$
U_2
0
n_g
U_3
$-z_g$
z
x
y
φ
$\langle U_l U_3^* \rangle$
$2k_0\Delta p_s$
$\langle U_2 U_3^* \rangle$
$\langle U_{l,eq} U_3^* \rangle$

Supplementary Figure S2. Axial-displacement sensitivity in reflection and single-transmission mode quantitative phase imaging. **(a)** Left panel: slab sample of mean refractive index $\bar{n}_s$ and thickness Δz placed in air, $n_a = 1$, at time $t = t_0$. Right panel: a minute thickness of δz etched out from the slab at time $t = t_1$. **(b)** Effect of detection noise on the phase $\varphi_{R/T}$ retrieved by reflection and single-transmission mode systems. The detection noise, $n_{\delta\varphi_{R/T}}$, is illustrated as a red vector restricted to a circle of radius σ. The noise can alter the phase $\varphi_{R/T}$ to within $\sqrt{\langle \delta\varphi_{R/T}^2 \rangle}$ on average.

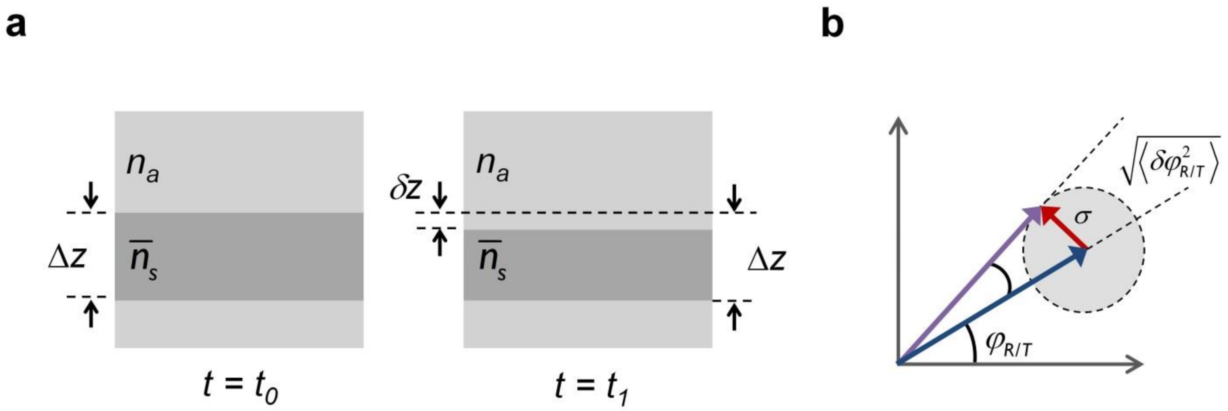

Supplementary Movie 1. Height dynamics of a water droplet on a ~170 μm-thick glass coverslip at room temperature across region I (outlined in Figure 5(a) by a dashed line) as imaged by the QPMES instrument with a 0.14-NA objective and a magnification of 7.5× at 5.25 phase-images per second. The height was computed by unwrapping the phase image data using the Goldstein's branch cut algorithm while referencing all time frames to a single identical region that was water-free over the entire course of the experiment.
